# The T2 Dark Spot Sign in Endometrioma

**DOI:** 10.5334/jbsr.2271

**Published:** 2020-12-01

**Authors:** Kristen Decommer, Frederik Feyaerts

**Affiliations:** 1 Ghent University, BE; 2 AZ Sint-Lucas, BE

**Keywords:** endometrioma, MRI, ovarian cyst, T2 dark spot sign, hemorrhagic cyst

## Abstract

**Teaching Point:** The T2 dark spot sign has low sensitivity but high specificity for endometrioma on MR imaging.

## Case Report

**Figure 1:**
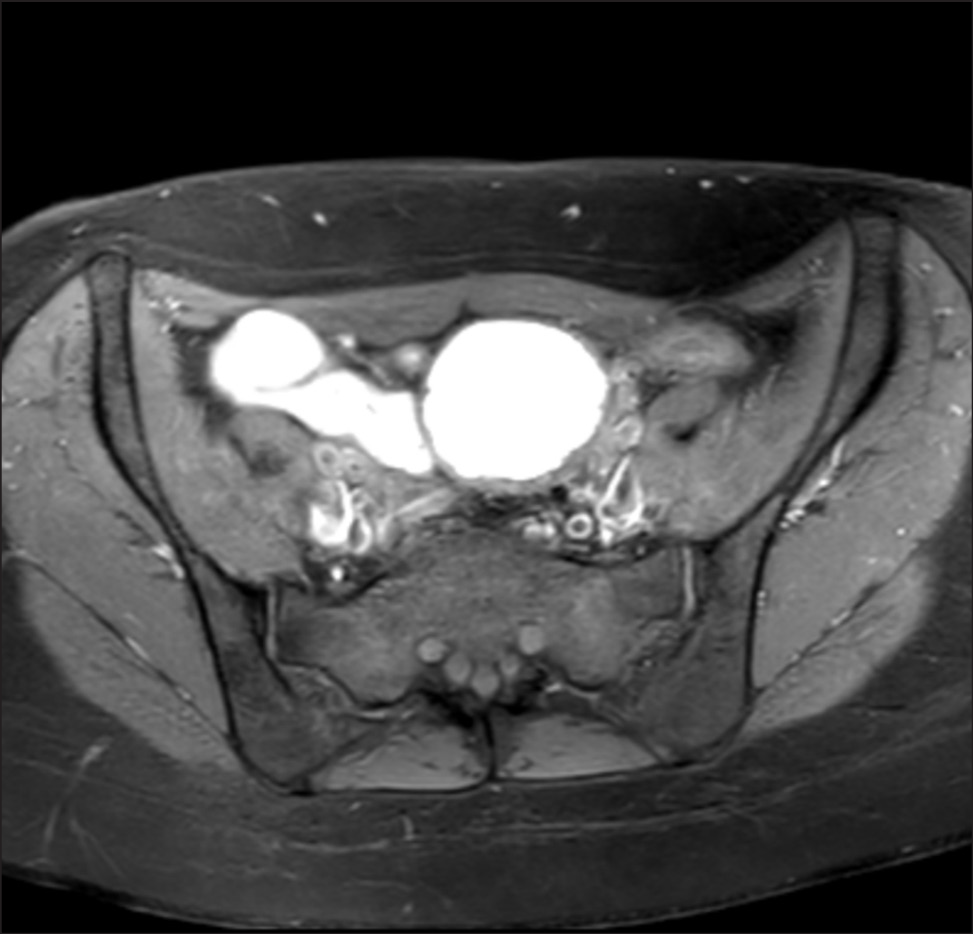


**Figure 2:**
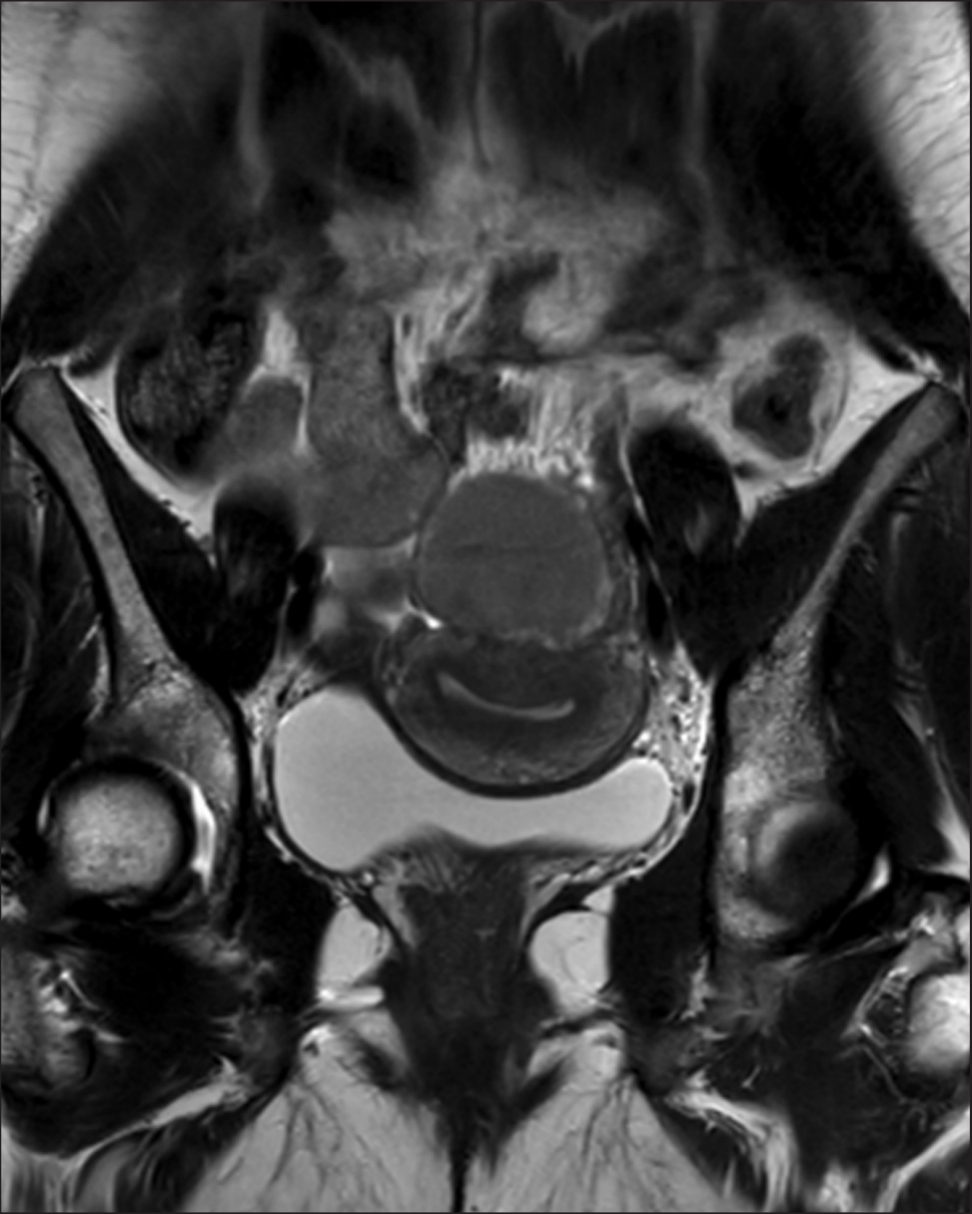


**Figure 3:**
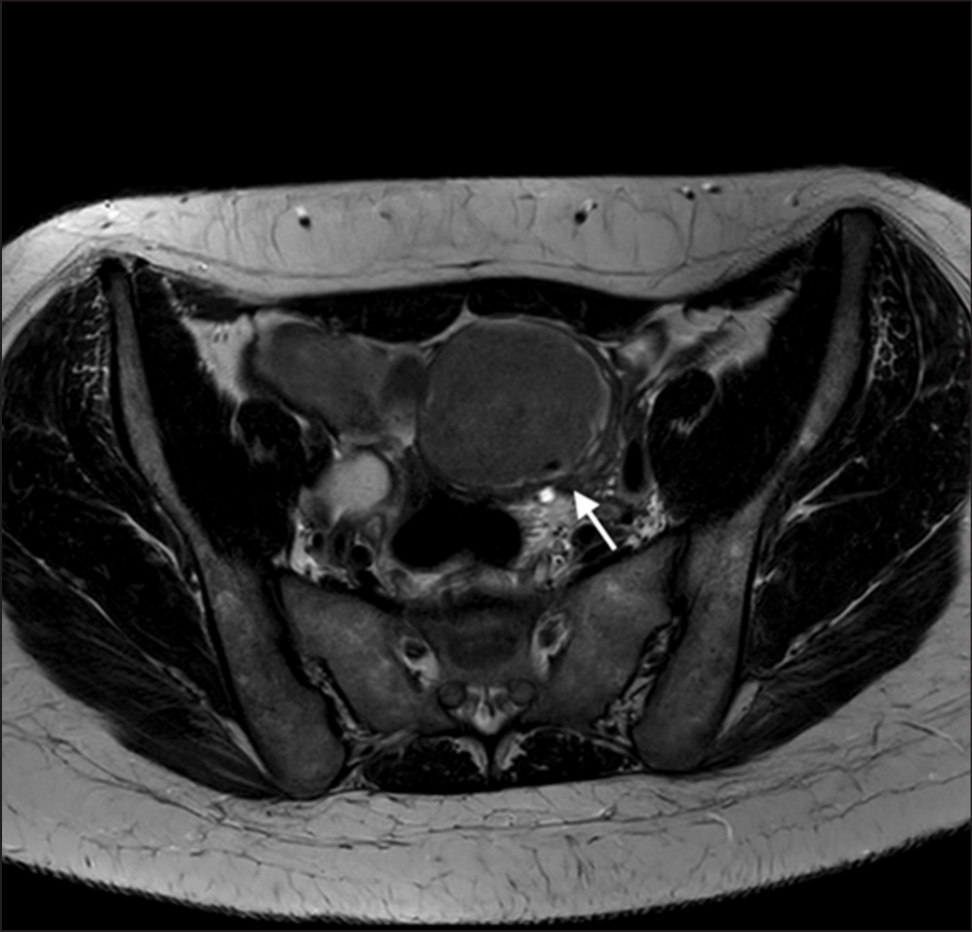


A 30-year-old nulliparous woman presented at the gynaecology department with dysmenorrhea and dyschezia. The patient was otherwise healthy and not using any medication. Transvaginal ultrasound (US) revealed a 6.5 cm echogenic left ovarian cyst. Subsequent magnetic resonance imaging (MRI) confirmed a thick-walled solitary left ovarian cyst with high signal intensity on fat-suppressed T1-weighted images (Figure 1), variable low signal intensity on T2-weighted images (Figure 2) and a focal signal void in the declive portion of the cyst (Figure 3). These findings are compatible with those of an endometrioma, confirmed by pathology after surgery.

## Comment

Endometrioma is a hemorrhagic ovarian cyst that arises from ectopic endometrial tissue. These lesions bleed cyclically. The repeated breakdown of red blood cells results in increased iron and protein concentrations.

MRI is an accurate imaging technique for diagnosing endometrioma. The high concentrations of degraded blood products in endometrioma result in T1 and T2 shortening effect, with subsequent high signal intensity on T1-weighted images and low signal intensity on T2-weighted images, or T2 shading. T2 shading has only a limited specificity for endometriomas (sens. 93%, spec. 45%) and cannot differentiate from functional hemorrhagic cysts with certainty. In contrast, the T2 dark spot sign has lower sensitivity, but a much higher specificity and does allow differentiation of functional hemorrhagic cysts (sens. 36%, spec. 93%) [1].

The T2 dark spot sign is defined as a focal hypointense spot that can appear anywhere within the cyst, but not in the cyst wall. It is thought to represent a retracted blood clot, containing heavy concentrations of protein and iron as a result of chronic hemorrhage, that causes the marked T2 shortening. The T2 dark spot sign should not be applied to lesions with solid components (possibly chronically hemorrhagic malignancies) and a small number of false positives have been reported in benign cystadenomas [1].
